# Exploring the Intrinsic Piezofluorochromic Mechanism of TPE-An by STS Technique

**DOI:** 10.1186/s11671-015-1036-7

**Published:** 2015-09-03

**Authors:** Shunyu Jin, Yan Tian, Fei Liu, Shaozhi Deng, Jun Chen, Ningsheng Xu

**Affiliations:** State Key Laboratory of Optoelectronic Materials and Technologies, Guangdong Province Key Laboratory of Display Material and Technology, Sun Yat-sen University, Guangzhou, 510275 People’s Republic of China

**Keywords:** TPE-An, STS technique, Piezofluorochromic, The HOMO-LUMO gap

## Abstract

9,10-bis(4-(1,2,2-triphenylvinyl)styryl)anthracene (TPE-An) materials have attracted considerable attention in recent years because they have high luminescence efficiency and excellent piezofluorochromic properties, which have potential applications in organic light-emitting display (OLED) area. Scanning tunneling spectroscopy (STS) technique was used to study the piezofluorochromic mechanism of aggregation-induced emission (AIE) materials for the first time. Photoluminescence (PL) experiments revealed that the emission peak of TPE-An is observed to exhibit a red-shift with the increase of the grinding time. A theoretical calculation was carried out to find the relationship between the bandgap of TPE-An and the external force by combination of the classical tunneling theory and STS results. It is found that when the pressure variation on the surface of TPE-An film was increased to be over 4.38 × 10^4^ Pa, the shrink of the highest occupied molecular orbital (HOMO)-lowest unoccupied molecular orbital (LUMO) gap can arrive at 1.1 eV. It is concluded that the piezofluorochromic behaviors of TPE-An should originate from the shrinking effect of the bandgap under external force. Moreover, this research method may shed light on comprehending and adjusting the piezofluorochromic characters of other AIE materials.

## Background

The luminescent behaviors of organic materials usually turn worse due to the aggregation-caused quenching (ACQ) effect [1], which blocks off their further applications. Their luminescent efficiency cannot be effectively improved until a typical aggregation-induced emission (AIE) material (silole molecule) was found to successfully overcome the ACQ effect in 2001 [2]. In AIE material family, tetraphenylethene (TPE) derivatives have gotten a rapid development in the last decade [3–8]. As an important member of AIE family, 9,10-bis(4-(1,2,2-triphenylvinyl)styryl)anthracene (TPE-An) is very particular because it possesses unique piezofluorochromic characters [8–21]. As a result, TPE-An has attracted considerable attention because it has potential applications in organic light-emitting display (OLED), photovoltaic cells, transistors, and solid-state storage [22–26]. In recent studies, the photoluminescence (PL) peak of TPE-An material was firstly found to have a significant red-shift after a series of grinding treatments [27], revealing that the external force may have much effect on the fluorescence property of TPE-An. But until now, the relationship among the external force, the bandgap, and the PL peak shift of TPE-An cannot be thoroughly understood, which makes it hard to control the piezofluorochromic performances. Therefore, it provides a new challenge to find the intrinsic piezofluorochromic mechanism of TPE-An.

Scanning tunneling spectroscopy (STS) technique is a very useful tool to measure surface electron’s local density of states (LDOS) [28–30], which can give the level position of the highest occupied molecular orbital (HOMO) and lowest unoccupied molecular orbital (LUMO) in the energy-band diagram. Most of all, the working performance of TPE-An under the electrostatic force in STS measurements is very similar to that of TPE-An under the mechanical force. Therefore, STS should be an ideal tool to in situ study the piezofluorochromic property of TPE-An under the external force.

In this work, the piezofluorochromic mechanism of TPE-An film was investigated by STS technique for the first time. Combined STS results with energy-band theory, the energy-gap diagrams of TPE-An before and after deformation are compared to find out the relationships among the external force, the bandgap, and fluorescence property of TPE-An. The possible piezofluorochromic mechanism is also discussed here.

## Methods

### The Synthesis Method of TPE-An Film

**Scheme 1:**
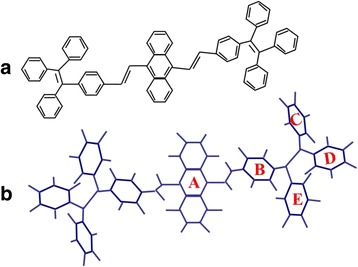
**a** Geometrical configuration of TPE-An molecule. **b** The conformer of TPE-An molecule with the lowest energy by simulation. This molecule has a highly twisted conformation. The bond angle (*A*-*B*) and (*B*-*C*) is 58°, and that of (*B*-*D*) is 80°. And the bond angle of (*B*-*E*) and (*C*-*D*) is equal to 75°

The known Wittig-Horner reaction route was adopted to synthesize the TPE-An powders (99 wt. %), and the detailed synthesis procedure is similar to that used in refs. [26, 30, 31]. Typical geometrical configuration of TPE-An molecule can be given by Gaussian 03 Revision program, as shown in Scheme 1. One can see in Scheme 1 that TPE-An possesses a highly twisted molecular structure. The fabrication process of TPE-An film can be depicted as follows. TPE-An (99 wt. %) powders were used as source materials and loaded into a tungsten boat. In the thermal evaporation process, Si (100) substrate was placed above the boat with a distance of about 20 cm. The growth pressure was kept at 4 × 10^−6^ mbar in the reaction process. After the evaporation lasted for 0.5–1 h, TPE-An film was found to deposit on the surface of Si substrate.

### Characterization

PL spectra of TPE-An samples under different grinding conditions were measured in air by the Shimadzu RF-5301PC spectrometer. The excitation wavelength was 365 nm in PL measurements. FTIR spectrometer (Thermo Fisher Scientific Inc, Nicolet 6700) was used to obtain the room temperature mid-infrared (Mid-IR) spectra of TPE-An, and the measurement wavenumber was ranged from 4000 to 1000 cm^−1^. And scanning tunneling microscope (STM) experiment was carried out in an ultrahigh vacuum (UHV) Omicron system. STM tips were prepared by electrochemical etching of a polycrystalline tungsten wire. The base pressure of STM chamber was 3 × 10^−9^ mbar. STS measurement was controlled by 7265 DSP lock-in amplifier electronics. The Kelvin probe force microscopy (KPFM) experiment was performed in the atomic force microscopy (AFM) system (Dimension FastScan, Bruker Corporation).

## Results and Discussion

**Fig. 1:**
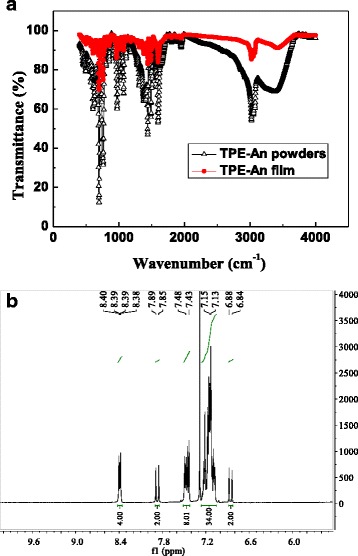
**a** Typical mid-infrared spectra of TPE-An powders and film. **b** Representative ^1^HNMR spectrum of TPE-An sample in CDCl_3_

To confirm the composition of the sample, Mid-IR spectra of the TPE-An source materials and the as-grown film are compared together, as shown in Fig. 1a. It is obviously seen that their peaks nearly coincide with each other. Moreover, Fig. 1b gives the representative [1] ^1^HNMR spectrum of the sample in CDCl_3_ to further reveal its composition. Combined the Mid-IR with [1] ^1^HNMR results, the as-prepared film can be proven to be TPE-An.

**Fig. 2:**
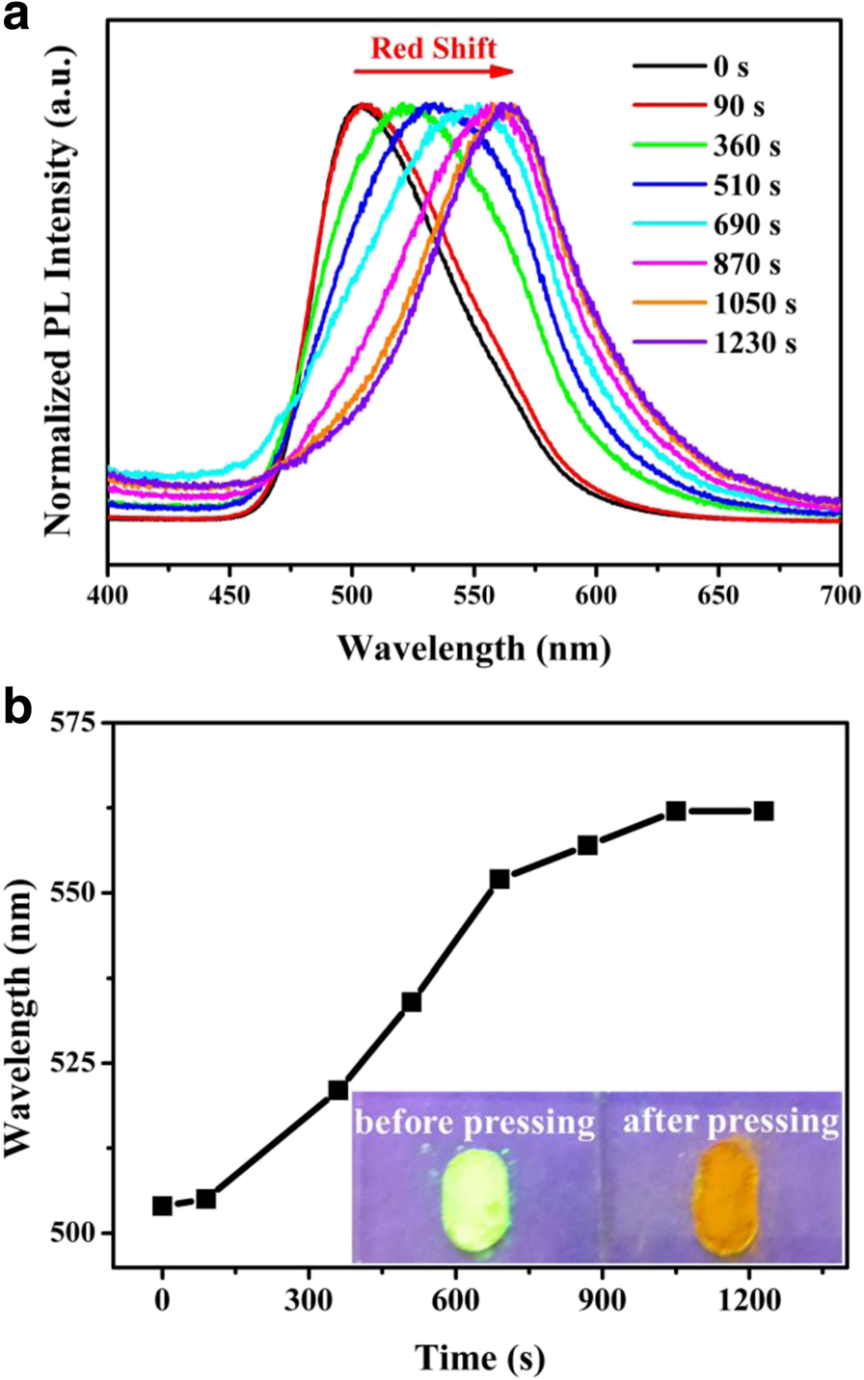
**a** Normalized PL spectra of TPE-An sample before and after grinding. **b** The curve of the grinding time versus the PL peak of TPE-An. The *inset* gives the emission image of TPE-An samples before and after pressing (1200 s), respectively

It is known that PL technique is usually used to investigate the piezofluorochromic property of AIE materials by varying the grinding time to obtain different external forces [27, 32, 33]. Here, it was performed on the as-prepared TPE-An samples. Typical PL spectra of TPE-An samples in different grinding times can be found in Fig. 2a. It is found that the PL peak of TPE-An before grinding is located at 504 nm. It is obviously seen that the emission peak of TPE-An exhibits a red-shift with the increase of the grinding time, which corresponds to the increase of the external force, as discussed in most references [27, 32, 33]. The maximum red-shift of the PL peak arrives at 58 nm when the grinding time increases to 1050 s, which suggests that the piezofluorochromic behaviors of TPE-An may have some relationship with the bandgap variation. One can see in Fig. 2b that when the grinding time is further lengthened, the emission peak almost remains unchanged. It is proposed that the polarization saturation of TPE-An molecules may be responsible for this experimental phenomena because the compressive force is bigger than the critical force in this situation. And the detailed mechanism is still under research. It is noted that the fluorescence of TPE-An changes from green into orange after a grinding time of 1200 s in the inset, which reveals a clear piezofluorochromic effect occurs when it suffers from the applied force.

In some reports [10, 33–40], piezofluorochromic property of AIE materials was suggested to be resulted from the substantial alternation of π-π overlap, the change of intermolecular packing modes or the transformation from amorphous state to crystalline state. Although these changes or transformations must lead to the bandgap variation of AIE to some extent, their energy-band diagram under the external force is seldom concentrated in recent studies. To find the intrinsic mechanism of TPE-An’s piezofluorochromic property, the classical energy-band theory in semiconductor physics is adopted in our research. Einstein’s photoelectronic equation gives the relationship between the HOMO-LUMO gap (*E*) and the emission light’s wavelength (*λ*), which can be written as:

1 $$ E=h\cdot \gamma =\frac{hc}{\lambda } $$

where the Planck constant *h* is 4.14 × 10^−15^ eV s and the light speed *c* is 3.0 × 10^8^ m/s. Based on Eq. (1), the photon’s energy is dependent on the HOMO-LUMO gap between the ground state and the excited state. According to Eq. (1), the wavelength discrepancy ∆*λ* can be deduced as:

2 $$ \varDelta \lambda =hc\left(\frac{1}{E_2}-\frac{1}{E_1}\right) $$

where *E*_1_ and *E*_2_ are respectively the HOMO-LUMO gap (bandgap) under two different external forces. Based on the abovementioned analysis, the emission peak of TPE-An should exhibit an obvious red-shift with the increase of the grinding time (external force), which coincides with our PL results (Fig. 2) well.

**Fig. 3:**
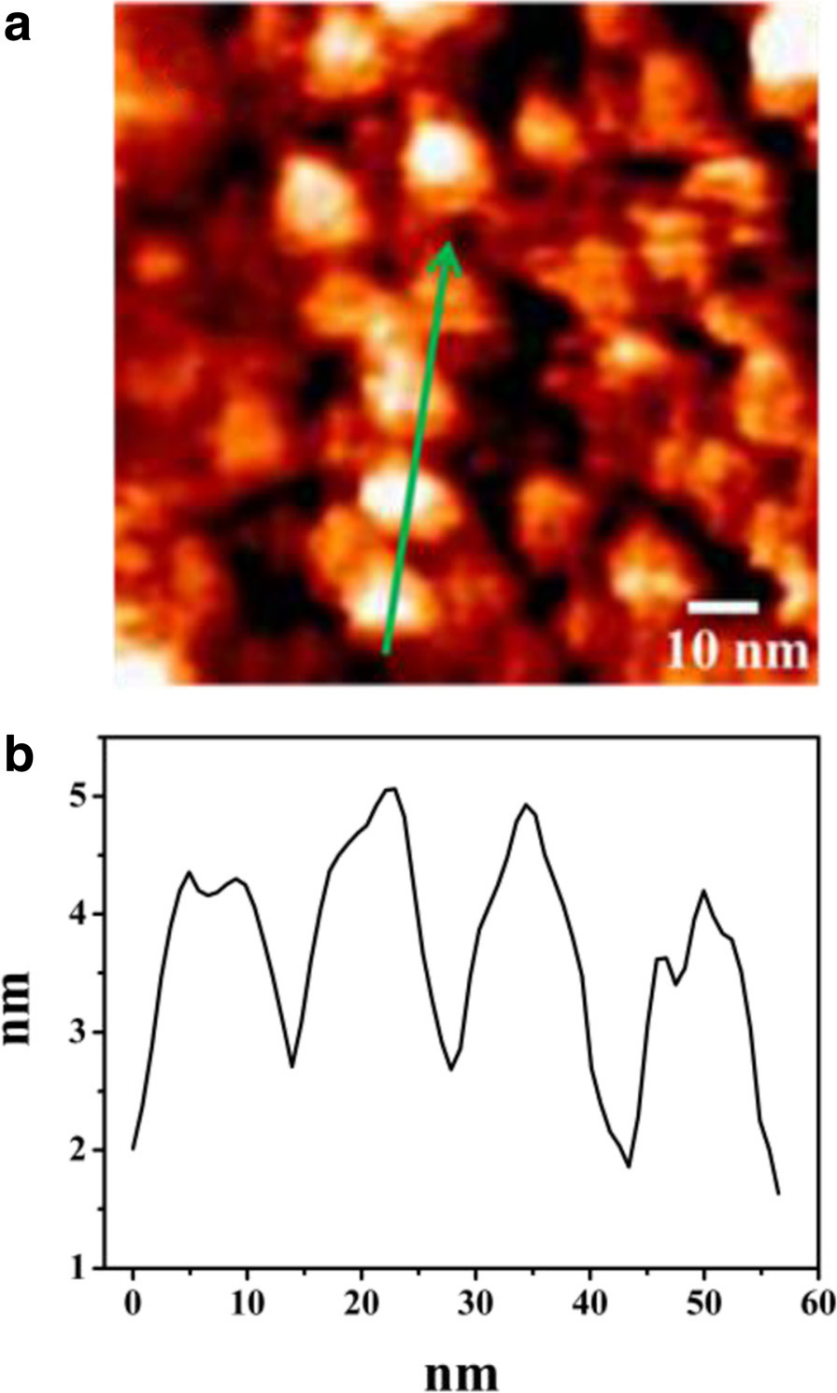
**a** Typical STM image of TPE-An film on Si (001) substrate (100 × 100 nm). The given voltage was −1.2 V, and the set current was 1000 pA. **b** The surface height distribution curve of TPE-An film, which corresponds to the cross-section curve in Fig. 2a

Although the PL spectra reveal that TPE-An possesses unique piezofluorochromic property, a quantitative relationship between the HOMO-LUMO gap and the external force cannot be worked out by this technique. In our study, STM technique is performed on TPE-An film to solve this question. Figure 3a is a 100 × 100 nm STM image of TPE-An film. Island structures with nanometer size are seen to uniformly distribute over the whole surface. Boundaries among the nanoisland structures are very distinct. Figure 3b provides the height distribution information of the sample’s surface, which corresponds to the cross-sectional arrow in Fig. 2a. It can be seen that the averaged diameter of nanoislands is about 20 nm and their height ranges from 2 to 3 nm. The smooth surface morphology of TPE-An film will benefit for the following STS measurements.

**Fig. 4:**
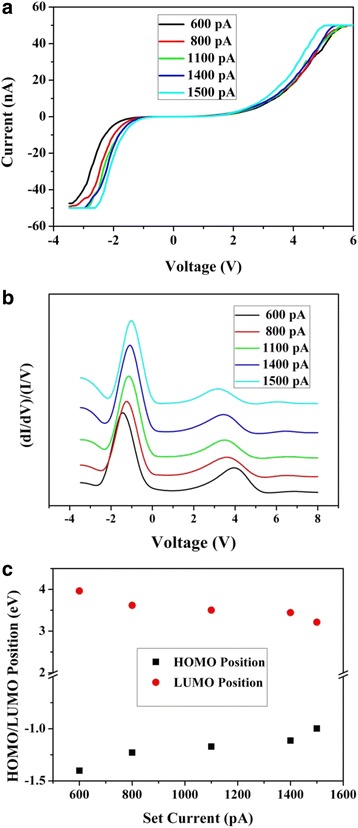
**a** *I*-*V* curves of TPE-An film on Si substrate. **b** Normalized conductivity versus voltage curves at different set currents. The given voltage was −1.2 V. The set currents were 600, 800, 1100, 1400, and 1500 pA, respectively. **c** The level positions of the HOMO and LUMO of TPE-An film at different set currents

To determine the surface energy-band diagram of TPE-An film, the *I*-*V* curves and their corresponding normalized differential conductivity versus voltage ((*dI*/*dV*)/(*I*/*V*)-*V*) curves are respectively given in Fig. 4a, b. In STS measurements, the applied voltage ranged from −3.5 to 6.0 V, and the set current *I*_set_ varied from 600 to 1500 pA. In Fig. 4a, the conductivity of TPE-An film is observed to vary with the set current *I*_set_. It is seen that the spacing between two zero-current offsets of the sample turns shorter with the increase of *I*_set_. Usually, the voltage variation between two zero-current offsets in *I*-*V* curve is defined as the HOMO-LUMO gap in STS study. Under this circumstance, the decrease of the spacing of two zero-current offsets corresponds to the shrink of the HOMO-LUMO gap of TPE-An. We propose the following explanations to comprehend these experimental results. A higher *I*_set_ suggests a smaller distance between the tip and film surface in constant-current working mode of STM. And the applied voltage keeps invariable at different set currents in measurements. As a result, the electrostatic force will increase with the increase of *I*_set_, which implies that a stronger compressive force imposes on the surface of TPE-An film at high current. Under the electrostatic force, the HOMO-LUMO gap of TPE-An film is observed to vary with *I*_set_ in STS experiments. By calculating the experimental results in Fig. 4b, the curve of the HOMO/LUMO position versus *I*_set_ is provided in Fig. 4c. The HOMO-LUMO gap is found to decrease from 5.42 to 4.31 eV when *I*_set_ increases from 600 to 1500 pA. It is noted that the maximum variation of the TPE-An’s gap reaches 1.11 eV when the set current increases to 1500 pA. It is suggested that the surface band diagram of TPE-An exhibits a significant variation under a large electrostatic force. Correspondingly, the maximum red-shift of PL peak is worked out to be 59 nm under electrostatic force based on Eq. (2). Therefore, it reveals that the stress-induced emission properties of TPE-An should originate from the variation of the HOMO-LUMO gap under the external force.

**Fig. 5:**
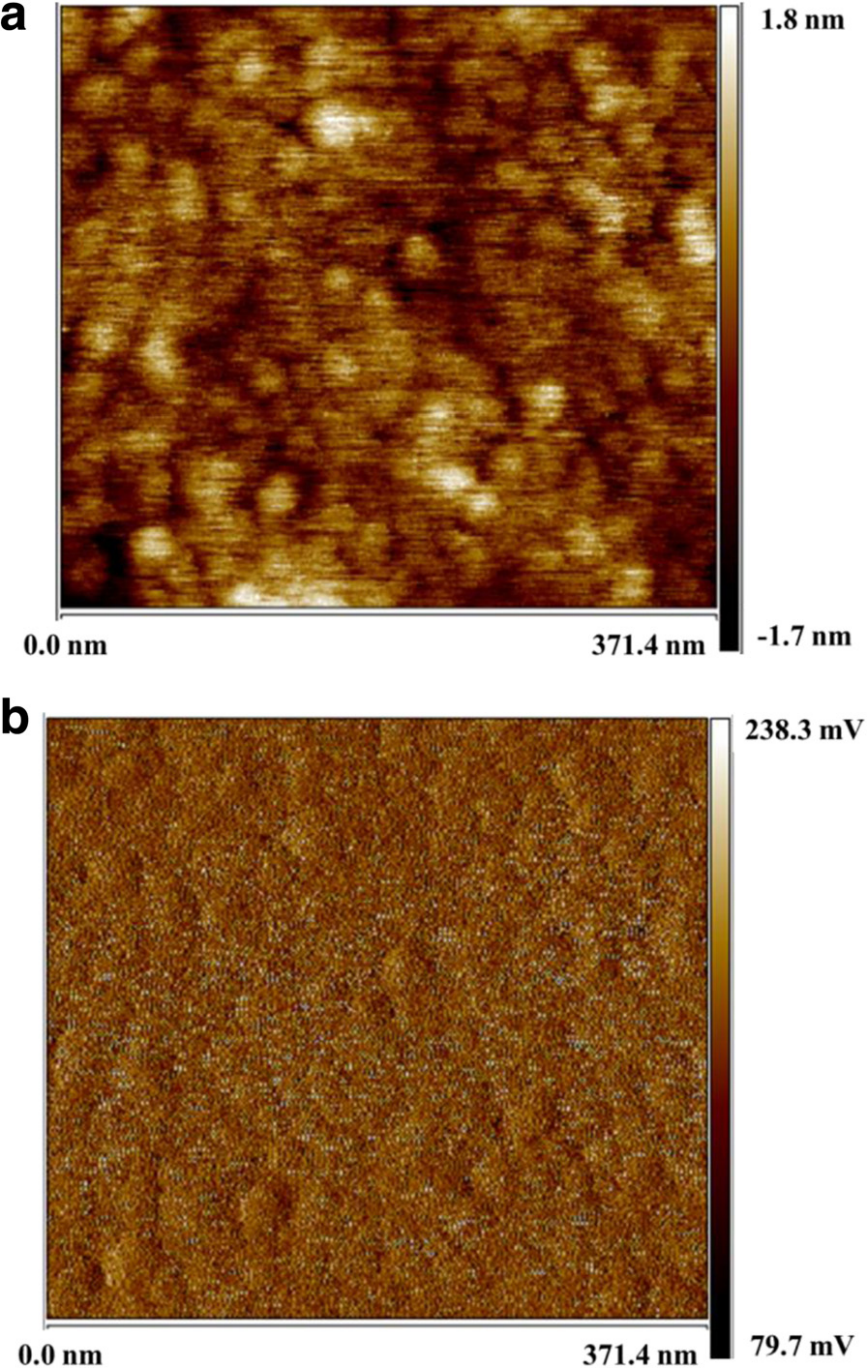
The AFM image (**a**) and the corresponding potential image (**b**) of TPE-An film (371.4 × 371.4 nm)

To calculate the electrostatic force between W tip and TPE-An’s surface in STS experiments, the surface work function of TPE-An film must be worked out firstly. Here, KPFM technique is used to obtain the work function TPE-An film by measuring the contact potential difference *V*_CPD_ between AFM tip and sample. *V*_CPD_ can be described as:

3 $$ {V}_{\mathrm{CPD}}=\frac{\phi_{\mathrm{sample}}-{\phi}_{\mathrm{tip}}}{-e} $$

where *e* is the electron charge, and *Ф*_tip_ and *Ф*_sample_ are respectively the work function of AFM tip and sample. It is necessary to get the work function of the AFM tip in KPFM measurements in advance according to Eq. (3). Firstly, *Ф*_tip_ is measured to be 5.14 eV by using the standard Au film (work function is 5.1 eV) as a reference [41, 42]. Subsequently, KPFM measurements were performed on TPE-An film by using the AFM tip with the known work function. Figure 5a, b provides the AFM image and the potential image of TPE-An film, respectively. The contact potential difference *V*_CPD_ is confirmed to be 0.16 ± 0.03 eV by analysis of Fig. 5b. Finally, the averaged surface work function of TPE-An (*Ф*_sample_) can be determined to be 4.98 ± 0.03 eV based on Eq. (3).

To quantitatively calculate the tip-surface electrostatic force, the classical tunneling theory is used in our study. According to this theory, the tunneling current can be expressed as [43]:

4 $$ I\propto {V}_b \exp \left(-\frac{2\sqrt{2m\phi }}{\hslash }d\right) $$

where $\phi =\frac{1}{2}\left({\varPhi}_{\mathrm{tip}}+{\varPhi}_{\mathrm{sample}}\right)=5.23\kern0.5em \mathrm{eV}$ is the averaged work function of STM tip and TPE-An film, *m* is the electron mass (9.1 × 10^−31^ kg), *ℏ* is $\frac{h}{2\pi }$, *V*_*b*_ is the applied voltage, and *d* is the tip-surface distance. Equation (4) can be also written as:

5 $$ d=\frac{1}{-\frac{2\sqrt{2m\phi }}{\hslash }} \ln I-C $$

where $C=\frac{h}{2\sqrt{2m\phi }} \ln \left(\frac{1}{T{V}_b}\right)$ is a constant for the same STM tip and sample. From Eq. (5), the tip-surface distance *d* decreases with the increase of the set current *I*_set_. Thus, the variation *Δd* of the tip-surface distance can be derived as:

6 $$ \varDelta d=-\frac{1}{\frac{2\sqrt{2m\phi }}{h}}1\mathrm{n}\frac{I_2}{I_1} $$

where *I*_1_ and *I*_2_ are two different set currents. In STS experiments, *I*_set_ is respectively 600, 800, 1100, 1400, and 1500 pA. The corresponding charge *q* on the surface area $\overrightarrow{S_0}$ of the tip can be worked out by the classical Gauss law:
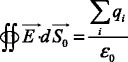


If the electric field *E* is assumed to be uniformly distributed between tip and surface, Eq. (7) can be simplified as $E{S}_0=\frac{q}{\varepsilon_0}$ because $\overrightarrow{E}$ is vertical to the Gauss surface in experiments. Therefore, the pressure *P* on the surface of TPE-An film can be expressed as:

8 $$ P=\frac{F}{S_0}=\frac{Eq}{S_0}={\varepsilon}_0{E}^2={\varepsilon}_0{\left(\frac{V_b}{d}\right)}^2 $$

where *ε*_0_ is the permittivity of the free space (8.85 × 10^−14^ C/V cm). According to Eq. (8), the pressure *P* of the electrostatic force *F* will increase with the decrease of the tip-surface distance *d*. In our calculation, the effective action area of the electrostatic force can be thought to be approximately equal to the tip-surface area *S*_*0*_. Combined Eq. (6) with Eq. (8), the pressure variation *ΔP* resulting from the current variation can be deduced as:

9 $$ \varDelta P=-2{\varepsilon}_0\frac{U^2}{d^3}\varDelta d={\varepsilon}_0\frac{U^2}{d^3}\frac{h}{\sqrt{2m\phi }}1\mathrm{n}\frac{I_2}{I_1} $$

where the tip-surface distance *d* is 0.6 nm in STS measurements [44]. Based on Eq. (9), both *Δd* and *ΔP* can be obtained, as summarized in Table 1. It is seen that the variation *Δd* of the tip-surface distance reaches 0.38 Å when *I*_set_ increases from 600 to 1500 pA. Moreover, the shrink of the HOMO-LUMO gap arrives at 1.11 eV when *ΔP* increases to 4.38 × 10^4^ Pa.

**Table 1:** A summarization table of *Δd*, *ΔP*, *ΔE*, and *Δλ* at different set currents by tunneling theory

	*ΔI*_21_	*ΔI*_31_	*ΔI*_41_	*ΔI*_51_
*Δd* (Å)	−0.12	−0.25	−0.35	−0.38
*ΔP* (Pa)	1.38 × 10^4^	2.90 × 10^4^	4.05 × 10^4^	4.38 × 10^4^
*ΔE*_*g*_ (eV)	−0.57	−0.81	−0.87	−1.11
*Δλ* (nm)	26.93	40.26	43.81	59.01

**Δ** *I*_21_ *= I*_2_ *− I*_*1*_, **Δ** *I*_31_ *= I*_*3*_ *− I*_*1*_, *∆I*_41_ *= I*_4_ *− I*_1_, *∆I*_51_ *= I*_5_ *− I*_1_

*d* is the tip-surface distance, *P* is the pressure on the sample’s surface, *Eg* is the HOMO-LUMO gap, and *λ* is the wavelength of PL peak

**Fig. 6:**
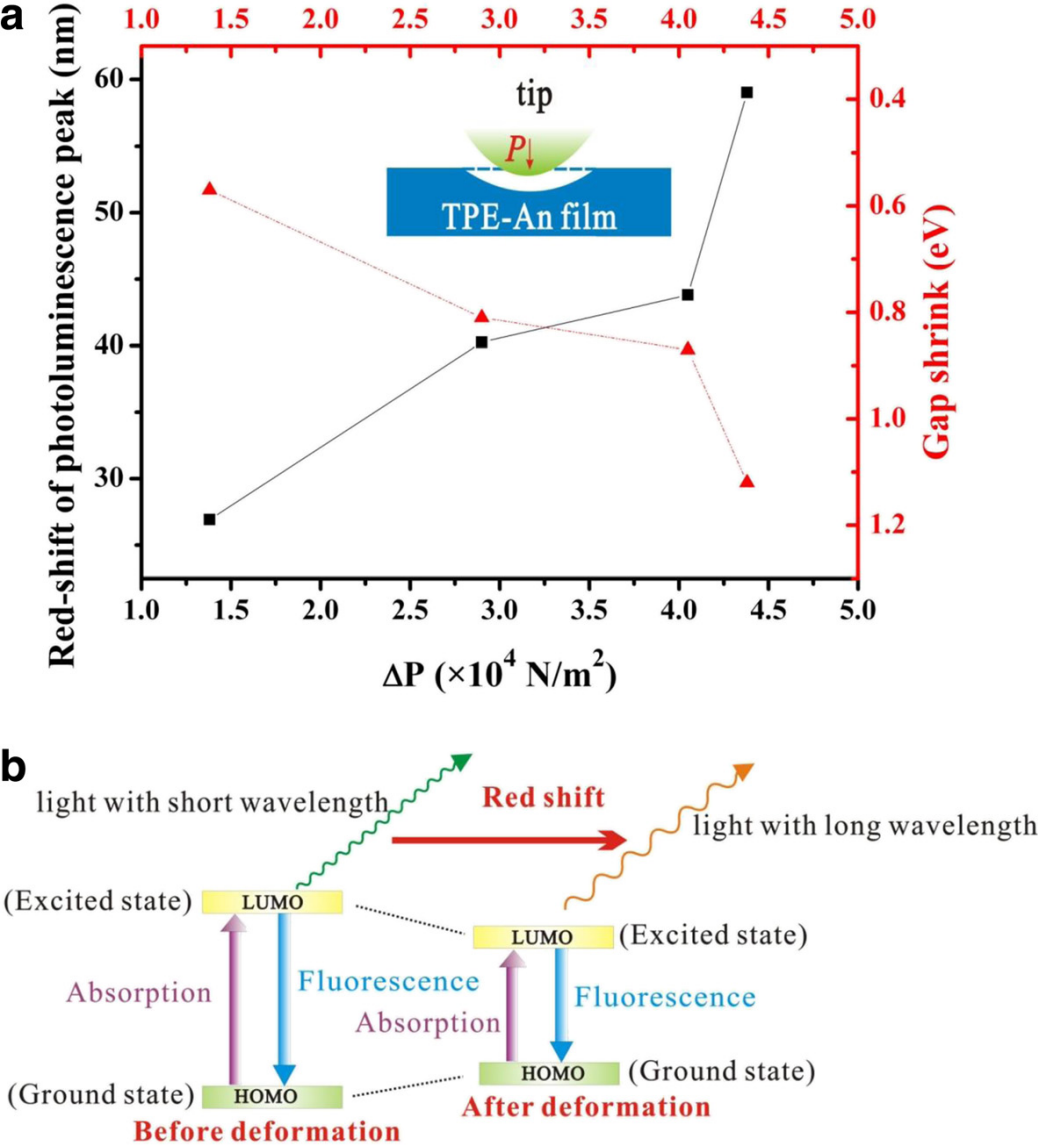
**a** The relationship among the red-shift of PL peak, the HOMO-LUMO gap variation, and the applied pressure variation. The *inset* is the surface deformation of TPE-An film suffered from the electrostatic force in STS experiments. **b** A schematic illustration of the piezofluorochromic behaviors of TPE-An materials by the band model

The relationship among the red-shift of PL peak, the HOMO-LUMO gap variation, and the applied pressure variation is shown in Fig. 6a. It is seen in Fig. 6a that the red-shift extent of the PL peak turns larger with the decrease of the HOMO-LUMO gap. It is also found that the red-shift extent of the PL peak will increase with the pressure of external force. To better understand the piezofluorochromic mechanism of TPE-An sample, the inset gives the schematic diagram of TPE-An sample suffered from the electrostatic force, in which one can see that an obvious deformation occurs on the surface under external force. Figure 6b gives the surface energy-band diagram of TPE-An under external force (compressive force or electrostatic force). As shown in Fig. 6b, the HOMO-LUMO gap of TPE-An decreases with the increase of the compressive force or the electrostatic force. Correspondingly, the wavelength *λ* of emission light turns larger with the shrink of the HOMO-LUMO gap, which causes the red-shift of PL peak in Fig. 1.

## Conclusions

In summary, STS technique was firstly applied to investigate the piezofluorochromic mechanism of TPE-An film. The relationship between the HOMO-LUMO gap variation and the tip-surface electrostatic force has been quantitatively obtained by combination of the tunneling theory and STS results. It is found that the shrink of the HOMO-LUMO gap arrives at 1.1 eV when the pressure increment on the surface of TPE-An film is over 4.38 × 10^4^ Pa, which can result in a clear red-shift (59 nm) of PL peak. Moreover, the HOMO-LUMO gap TPE-An has exhibited the same shrinking tendency with the increase of external force in both STS and PL measurements. Therefore, it is suggested that the gap-shrinking effect of TPE-An under external force may be responsible for its piezofluorochromic behaviors. And this research method may give a helpful reference for comprehending and modulating the piezofluorochromic behaviors of other AIE materials.
